# Production and Utility of Extracellular Vesicles with 3D Culture Methods

**DOI:** 10.3390/pharmaceutics15020663

**Published:** 2023-02-16

**Authors:** Mar Casajuana Ester, Richard M. Day

**Affiliations:** Applied Biomedical Engineering Group, Centre for Precision Healthcare, UCL Division of Medicine, University College London, London WC1E 6JF, UK

**Keywords:** extracellular vesicles, 3D culture, scaffolds, bioreactors, spheroids

## Abstract

In recent years, extracellular vesicles (EVs) have emerged as promising biomarkers, cell-free therapeutic agents, and drug delivery carriers. Despite their great clinical potential, poor yield and unscalable production of EVs remain significant challenges. When using 3D culture methods, such as scaffolds and bioreactors, large numbers of cells can be expanded and the cell environment can be manipulated to control the cell phenotype. This has been employed to successfully increase the production of EVs as well as to enhance their therapeutic effects. The physiological relevance of 3D cultures, such as spheroids, has also provided a strategy for understanding the role of EVs in the pathogenesis of several diseases and to evaluate their role as tools to deliver drugs. Additionally, 3D culture methods can encapsulate EVs to achieve more sustained therapeutic effects as well as prevent premature clearance of EVs to enable more localised delivery and concentrated exosome dosage. This review highlights the opportunities and drawbacks of different 3D culture methods and their use in EV research.

## 1. Introduction

Extracellular vesicles (EVs) are lipid-bound vesicles which are important mediators in intercellular communication and which are secreted by most eukaryotic and prokaryotic cells, including animal cells and bacteria [1,2]. The presence of EVs in the extracellular space was identified in 1980 [3]. However, the secretion of EVs was initially seen as a form of secreting unwanted proteins or cellular waste resulting from either cellular damage or homeostasis [4,5]. In the mid-1990s, EVs were shown to induce vital immune responses, and in the last decade, with the advance of research methods and techniques, numerous studies have identified EVs as significant mediators in cell–cell communication [6]. EVs therefore play a fundamental biological role in cellular interactions in health and disease [1]. EVs are heterogenous vesicles and are classified based upon their biogenesis, contents, and sizes. Exosomes are lipid-bilayer-enclosed small EVs that originate from the endosomal plasma membrane [6,7,8]. Given their roles as vesicles in intracellular communication, EVs are involved in many physiological processes. This characteristic, together with their molecular composition, which reflects the status of the secreting cell, gives EVs the capacity to provide great clinical potential. There is a large number of ongoing pre-clinical and clinical studies evaluating the safety and efficacy of EVs for use as drug delivery agents, therapeutics, and novel tools for diagnostics [9,10,11]. EVs have uniquely high targeting capabilities and many studies have demonstrated both their autocrine and paracrine effects. Cancer cells, for example, can interact with each other through EV-supported cell–cell communication, promoting metastasis. Further concerning their naïve targeting capabilities, it is also possible to engineer EVs (e.g., with a particular type of antibody) to alter their targeting behaviour [12]. EVs also have lower immunogenicity than the cells they are derived from, offering a great opportunity for the improvement of current therapeutic systems [11]. An example of this is the use of EVs in cardiovascular diseases. Given their capability to transport cell–cell transferring proteins and messenger RNAs (mRNAs) amongst others, they have been proven to have pro-angiogenetic, pro-coagulant and anti-inflammatory effects as well as the ability to modulate cardiomyocytes and endothelial cells, inducing blood vessel formation and restoring oxygen supply to hypoxic regions [8].

EVs play a key role during cancer and tumour growth. EVs released from cancerous cells have been shown to promote tumour growth by promoting cell proliferation, angiogenesis, increasing the migratory and invasive capacity of cancerous cells that contribute to metastatic lesions, and increasing resistance to certain drugs [13,14]. They may therefore provide disease biomarkers suitable for non-invasive, detailed disease prognosis and diagnostic tools [15]. Several studies have shown that certain mRNAs and proteins are upregulated in EVs derived from cancer cells and could therefore be used as biomarkers for a range of malignancies including ovarian, breast, cervical, gastric, pancreatic, and lung cancers. EVs can be found in a variety of body fluids, such as plasma, saliva, and urine, and can therefore be used as disease biomarkers [16]. Furthermore, EVs are highly attractive as drug delivery agents due to their low toxicity, low immunogenicity, and ability to penetrate tight boundaries, such as the blood–brain barrier (BBB) [17]. The unique capacity to cross the BBB makes EVs very attractive carriers for carrying therapeutic drugs and genes to treat neurologic and psychiatric disorders [18].

The methods used for the isolation and purification of EVs must be standardised to ensure EV production is consistent and reproducible. Common isolation techniques, which are discussed later in the review, are often challenging for scaling up production. However, in recent years, new technologies—such as microfluidics, which are also discussed later in the review—have emerged as promising tools for EV research, offering higher EV yields and purities [19]. This review focuses on research studies performed on all EV subtypes, with a particular emphasis on exosomes, which are the most widely studied EV subtype in clinical and pharmacological fields. Where reviewed articles refer to research conducted specifically on exosomes, this has been stated. Where the articles refer to EVs, this may include exosomes or other types of EVs, such as microvesicles (MVs).

Studies are also focusing on enhancing the therapeutic potential of EVs as well as on strategies to achieve their sustained release. Three-dimensional (3D) culture systems are increasingly recognised as tools in bioprocessing and biomedical research. Three-dimensional cell culture consists of creating an in vitro environment that mimics the native tissue. This approach may consist of culturing a single cell type or co-culturing multiple cell types [20]. Culturing cells in 3D enables cells the formation of cell–cell and cell–extracellular matrix (ECM) interactions not achievable with two-dimensional (2D) culture systems. This is suggested to improve control of cell physiology, such as cell morphology, proliferation and differentiation, response to stimuli, drug metabolism, and protein synthesis [21,22]. Three-dimensional cell culture can be achieved by either encouraging cell–cell contact so that cells form only cell–cell interactions (e.g., with spheroids) or by providing an exogenous matrix material that cells can attach to (e.g., scaffolds) [23]. Several studies have used 3D culture models to study EVs as drug carriers. Nooshabadi et al., for example, loaded exosomes obtained from endometrial cells with atorvastatin and assessed their activity in the growth of glioblastoma spheroids [24]. Three-dimensional cell culture systems have also been used to investigate EVs as novel biomarkers. EVs secreted from 3D systems differed in molecular signalling and protein profiles compared to EVs secreted from 2D cultures [23,25]. Three-dimensional culture models have also been used to study the therapeutic effect of EVs, and several studies have shown that EVs secreted from 3D cultures have enhanced therapeutic effects, such as superior anti-inflammatory and pro-angiogenic effects [26,27,28]. Finally, 3D culture methods enable the growth of larger quantities of cells via more space- and cost-efficient methods, translating to a larger EV production, which is necessary to clinically translate EV-based therapies [20].

## 2. Subtypes of Extracellular Vesicles

EVs are heterogenous populations of lipid-bound vesicles and are naturally secreted by cells into the extracellular space. They can broadly be divided into four main subtypes—exomeres, exosomes, MVs, and apoptotic bodies—characterised based upon their biogenesis, release pathways, sizes, contents, and functions (Table 1) [29].

## 3. Exosomes as a Subset of Extracellular Vesicles—Their Origin and Characteristics

**Figure 1:**
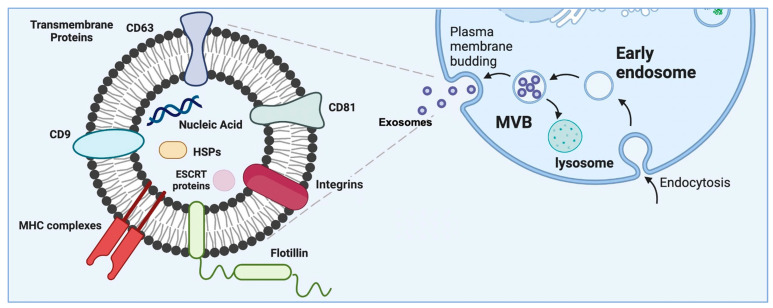
Biogenesis and main components of exosomes.

The biogenesis of exosomes starts with endocytosis, a process which consists in the inward budding of the cell membrane to form endosomes, also known as intraluminal vessels (ILV). As part of this, nucleic acids, proteins, and lipids are wrapped into ILV [3,31]. These early endosomes mature into late endosomes as a result of their membrane undergoing a series of inward invaginations that subsequently close [3]. Late endosomes are also known as multivesicular bodies (MVB), and these contain multiple vesicles, each enclosing a small part of the cytosol, including various proteins and nucleic acids [4]. MVB can then fuse with lysosomes, which cause them to degrade, or alternatively with the plasma membrane, releasing exosomes into the extracellular space (Figure 1) [3,4,31].

The processes by which ILV are loaded with a particular cargo and by which MVB release their content are not yet fully understood [29]. To date, researchers have found several pathways involved in the biogenesis of exosomes [31]. The endosomal sorting complexes required for transport (ESCRT) pathways play a prominent role in the biogenesis and secretion of exosomes [3,36]. Additionally, SNARE proteins and their effectors, such as RAB GTPases, have also been shown to have a major impact on exosome secretion [29]. By delivering these cargos to neighbouring cells, they can reprogram them, and therefore, exosomes play a role in vital cellular processes, such as immune responses, signal transduction, and antigen presentation [3].

Exosomes are enclosed in a lipid bilayer, and their characteristics are dependent on the cells that secrete them. The conditions of the cells secreting them (such as cellular health status and the microenvironment the cells are grown in) [1] are reflected in the exosomal cargo, comprising proteins and lipids as well as nucleic acids [37]. Proteins, such as receptors, transcription factors, and enzymes, can reflect the physiological status of a cell and are involved in regulating essential cellular processes. An example of this is exosomal protein transfer between cells, which can contribute to the spread of aggressive-phenotype cancer cells within heterogenous tumours [38].

Exosomes contain annexins; tetraspanin proteins (CD9, CD63, and CD81), which act as exosomal markers; ESCRT proteins; actins; and integrins (Alix and TSG101), important for cell communication. They also contain heat shock proteins (Hsp60, Hsp70, and Hsp90), which are involved in the folding and unfolding of proteins and in the protection of infectious diseases [39], as well as flotillins, which are involved in endocytosis, intracellular transport, and signalling [40]. Proteins such as heat shock proteins, CD63, and ESCRT are found in all exosomes, however, some of the proteins found in exosomal membranes reflect the cells they are derived from. For example, CD80 and CD86 are expressed on dendritic-cell-derived exosomes, and CD19 is expressed on B-cell-derived exosomes and as such, they can be used as disease biomarkers [36,41]. Additionally, antigen-presenting cells secrete EVs, expressing major histocompatibility peptide complexes (MHC) that induce specific T cell responses [42]. As such, exosomes carrying MHC-peptide complexes could promote adaptive immune responses by partially or fully activating helper and cytolytic T cells to kill tumour cells [43].

Exosomes also have a lipid composition, leading to characteristics which are specific to their cellular source. Exosomal lipid components include cholesterols, sphingolipids, phosphoglycerides, and ceramide (sometimes used to differentiate exosomes from lysosomes). The outer surface of exosomes is rich in saccharide chains, such as mannose, polylactosamine, alpha-2,6 sialic acid, and N-linked glycans [44]. These lipids have an effect in the exosomal sorting of cargo, their secretion, their structure, and signalling between exosomes and cells [45]. Exosomes, similarly to liposomes, have a membranous lipid bilayer which protects the exosomal cargo [29]. Additionally, exosomes contain a complex of nucleic acids. MicroRNAs (miRNAs) are the most abundant RNA species in exosomes [3]. They play a role in several biological processes such as exocytosis, haematopoiesis, and angiogenesis and are known to regulate cell–cell communication. Other exosomal RNA species include ribosomal RNA (rRNA), which differs from cellular RNA in that it makes up over 95% of the human transcriptome [46].

Internalisation of exosomes by target cells makes them attractive for use in disease diagnosis, drug delivery, and as therapeutic modulators.

### 3.1. EVs in Diagnostics

As mentioned previously, exosomal cargo reflects the cellular origin and physiological state of the donor. EVs derived from tumour, stromal, and immune cells contribute to the multiple stages of tumour growth, signalling, and progression as well as to resistance to therapy [43]. Hence, EVs have the potential to be used as disease biomarkers [47]. EVs have been found and isolated from a variety of body fluids, including blood, saliva, and amniotic fluids and therefore offer a non-invasive method of diagnosing patients and of monitoring their response to cancer immunotherapy [48].

The use of EVs could allow for earlier detection of several cancers [49]. Composition and concentration of exosomal miRNAs vary between diseased and healthy individuals [16]. For example, exosomal miR-21 has been reported to correspond with tumour progression and aggressiveness, making it possible to distinguish between patients with oesophageal squamous cell cancer from patients who have benign diseases without systemic inflammation [50]. Munagala et al. isolated and characterised exosomes from conditioned media of lung cancer and normal bronchial epithelial cells and found that 77 miRNAs were significantly modulated in lung cancer cells compared to healthy epithelial cells. miR-21 and miR-155 were found to be significantly upregulated in recurrent tumours compared to primary tumours [51]. Machida et al. studied the salivary exosomes from patients with pancreatic cancer and demonstrated that the levels of certain miRNAs were higher than those in healthy control patients [52]. Exosomal miRNAs have also been studied as potential markers to predict patients’ therapeutic responses to cancer treatments. Yuwen et al. demonstrated that increased levels of exosomal miR-146a-5p correlated with a better response to cisplatin, a common chemotherapy drug used to treat a variety of cancers [53,54].

EV proteins are also being studied as potential biomarkers for cancers. Exosomes containing CD24, EDIL3 and fibronectin proteins can be used to detect early breast cancer [55]. Additionally, exosomes containing CD24 Ala/Val single nucleotide polymorphisms (SNPs) genotype are associated with fast progressing autoimmune diseases and can therefore be used to detect this [56]. Additionally, exosomal PD-L1 expression levels are significantly correlated with tumour PDL-1 levels and can therefore be used as tool to evaluate the clinical efficacy of lung cancer immunotherapy [53,54,57]. Hoshino et al. demonstrated that exosomal integrins α6β4 and α6β1 were associated with lung metastasis and that exosomal integrin αvβ5 correlates to liver metastasis [58]. The same research group investigated the proteomic profiles of EVs and particles (EVPs) in human samples from tissue explants, plasma, and other bodily fluids and found that EV-specific proteins, such as VCAN, TNC, and THBS2, could distinguish tumours from normal tissues and could be used to determine the cancer’s origin [59].

Furthermore, EVs can be found in cerebrospinal fluid (CSF) and have been found to cross the BBB, emerging as novel biomarker sources to study neurodegenerative diseases [60,61]. For example, exosomes found in amyloid beta (Aβ) plaques, characteristic of Alzheimer’s disease, have been found to contain specific proteins, suggesting that they could serve as indicators of such [49]. Additionally, Ebrahimkhani et al. demonstrated that miRNAs associated with exosomes found in CSF can be used to diagnose multiple sclerosis (MS) [62].

Another condition for which EV research could be beneficial is diabetes. It was recently revealed that plasma-derived exosomes from patients with type 1 diabetes express a distinct miRNA signature [63]. Additionally, EVs have been associated in the pathogenesis of type 2 diabetes and could therefore be used as biomarkers for such [64]. EVs can be taken up by hepatocytes, muscle cells and other insulin target cell types and can cause an alteration in insulin signalling activation leading to disrupted normal metabolic responses of recipient sites to insulin causing insulin resistance [65]. An example of this is shown by hepatic EVs which contain miR-130a-3p, which attenuates glucose and lipid intolerance in adipose tissues by supressing the PHLPP2 gene [66]. Another example of this is miR-26a found in pancreatic β-cell-derived EVs, which decreases in obese animals, causing an increase to insulin resistance [67]. EVs can be used to detect multiple cardiovascular diseases such as atherosclerosis, myocardial ischaemia, cardiac fibrosis and ischaemia-reperfusion injury [68,69]. For example, Zampetaki et al. demonstrated that in patients with subsequent myocardial infarction, the expression of miR-126 in exosomes increased and could therefore be used as a biomarker for vascular injury [70].

The use of EVs as diagnostic markers is challenging in part because there are few unique cell-specific proteins and because EVs are able to circulate around the body, making it difficult to determine their tissue of origin [49]. There is a large number of non-EV particles, such as lipoproteins and protein complexes, in clinical samples. This could mean that biological effects and biomarkers assigned to EVs in some studies could be caused by contaminant constituents, which may interfere with EV-based biomarker discovery [71].

### 3.2. Therapeutic Application of EVs

Cells isolated from adult tissues, such as mesenchymal stem cells (MSC) have the ability to inhibit cell apoptosis, promote angiogenesis, and trigger cells that have survived in damaged tissues to proliferate [72]. Similar effects have been observed with the delivery of EVs secreted by MSC. One of the advantages of using EVs over their cells of origin is that cells can be subject to apoptosis and abnormal differentiation caused by stress during cell transplantation. Additionally, transplanted cells are at risk of immune rejection and do not possess the ease of targeting capabilities of EVs that enables them to target specific tissue areas [73]. As such, the therapeutic benefit of EVs is currently being explored in cancers, neurodegenerative diseases, immune-mediated conditions, and a variety of other fields [74,75].

Currently, a large number of stem cell research focuses on MSC due to their ability to replicate, differentiate into several cell lineages, and to participate in immunomodulation [76]. Research has explored the use of the paracrine signalling properties of MSC-derived EVs to treat and ameliorate a multitude of conditions. Yin et al. demonstrated that adipose-derived MSC (AD-MSC)-secreted exosomes led to a marked increase in H9C2 cell viability under hypoxic conditions in vitro and protected ischaemic myocardium from myocardial ischaemia reperfusion injury in vivo [77]. Another example of the therapeutic potential of MSC-derived EVs was demonstrated by Wang et al. Their studies demonstrated that exosomes secreted by bone marrow-derived MSC (BM-MSC) and AD-MSC cause superior cardioprotection compared to the cells they are derived from [78]. Another investigation demonstrated that BM-MSC-derived EVs promoted functional recovery of injured sciatic peripheral nerves using a rat model by improving nerve regeneration and increasing the expression of GAP-43, a marker for axon regeneration. [79]

In 2022, there were 33 registered exosome therapy clinical trials, of which 20 made use of MSC-derived exosomes [80]. Furthermore, biotechnology companies are developing MSC-derived exosomes to treat Alzheimer’s disease (Celltex Therapeutics, Houston, TX, USA), neural-stem-cell-derived exosomes for penetrating the blood–brain barrier (Aruna Bio, Athens, GA, USA), and an exosome-based vaccine platform to prevent infectious diseases (Codiak BioSciences, Cambridge, MA, USA) [81].

### 3.3. EVs as Drug Carriers

Compared to synthetic drug carriers, EVs isolated from a patient’s own cells offer higher biocompatibility and lower toxicity. EVs can penetrate tissues and cross tight boundaries, such as the BBB, making them attractive drug delivery vehicles. Additionally, it is possible to chemically modify the surface proteins found on EVs, increasing tissue specificity [17,82]. As a result, the first clinical trials in which EVs were used as drug delivery systems have already been reported [72]. The first clinical trial started in 2011 and investigated the ability of plant exosomes to more effectively deliver curcumin—a natural hydrophobic phenol extracted from turmeric—which has anti-inflammatory, anti-neoplastic, and anti-cancer properties to normal colon tissues and colon tumours [83].

Several research studies have also demonstrated the benefits of using EVs as drug carriers. For example, Kim et al. used exosomes to encapsulate the anti-cancer drug agent doxorubicin (Dox). Prolonged exposure to Dox caused significant cytotoxicity, limiting its prolonged use. Dox-loaded exosomes, on the other hand, were easily taken up by cells compared to unencapsulated Dox and Dox encapsulated by liposomes, reducing cytotoxicity [84]. Martins-Marques et al. demonstrated that the presence of the gap junction protein connexin43 (Cx43) in Dox-loaded EVs reduced the toxicity of the drug [85].

Additionally, several researchers have evaluated the exosomal delivery of paclitaxel (PTX), another commonly used anti-cancer agent. Kim et al., for example, showed that exosomes secreted by macrophages loaded with PTX showed higher anticancer efficacy in a mouse model of pulmonary metastases. They also demonstrated that it is possible to engineer these exosomes, leading to superior structures and higher therapeutic indices [86]. In another study, exosomes derived from human BM-MSC (hBM-MSC) were loaded with PTX, and their effect on triple negative breast cancer (TNBC) cells was investigated. Both in vitro and in vivo results showed significant tumour growth inhibition [87].

Li et al. demonstrated that exosomes can be used to deliver gemcitabine, the first-line chemotherapeutic drug for pancreatic cancer treatment. Results showed that exosomes loaded with gemcitabine supressed tumour growth with prolonged survival and caused minimal damage to normal tissues [88]. Another study showed that dopamine-loaded exosomes show a much better therapeutic effect in a Parkinson’s disease (PD) mouse model and a lower systemic toxicity when compared to free dopamine, which cannot cross the BBB, following intravenous administration [89].

There are, however, a few limitations to this field and challenges that still must be overcome. A major limitation is the lack of standardised techniques for the isolation and purification of EVs, which leads to impure and low EV yields as well as variation between different groups when studying the drug delivery potential of EVs [49,90]. Additionally, due to variation between EVs derived from the same cell type, the drug delivery potential remains unknown [49]. The different exosome isolation techniques are described later in this review. Briefly, these include common isolation techniques, such as differential centrifugation (dUC), density gradient centrifugation, ultrafiltration (UF), precipitation, size exclusion chromatography (SEC), and immunoaffinity capture, and emerging technologies, such as microfluidics. They each comprise a unique set of advantages and disadvantages [19,68,91]. One of the main challenges involving EV isolation is the presence of abundant protein mixtures which can be co-isolated with EVs. Additionally, these can coat EV membranes, influencing their recovery yield and biological activity [92]. In order to clinically translate EV research, the isolation method should be robust, easy-to use, economical, reproducible, time-saving, and high-throughput [93]. Common isolation methods do not meet these criteria, and significant efforts, including the development of microfluidics for EV isolation and membrane-based separation in which exosomes are isolated by binding their phosphate groups to some metal oxides, are being combined to achieve large-scale EV GMP production [19].

Another challenge involving drug-based EV delivery involves efficiently loading drugs and therapeutic cargoes into EVs. Loading methods can be mainly divided into two categories. The first category, passive loading, involves incubating the EVs with drugs; the drugs diffuse into the EVs along a concentration gradient, the cells they are derived from are treated with a drug so that they secrete EVs which are loaded with the drug. These methods tend to have low loading efficiencies and make it difficult to control the amount of drug being loaded into the EVs [94,95]. Additionally, the drug may be cytotoxic to the treated cells and may influence the EVs secreted. Pascucci et al. treated SR4987 MSCs with PTX. The isolated PTX-loaded EVs demonstrated strong anti-proliferative activity against CFPAC-1 human pancreatic cells in vitro. However, when loading PTX onto SR4987 MSC, ~15% of them underwent apoptosis due to PTX cytotoxicity [96]. The second category involves active cargo loading methods. These include sonication, electroporation, extrusion, freeze/thaw cycles, saponin pretreatment, and incubation with membrane permeabilisers amongst others [93]. Each of these have different loading capacities which will vary depending on the hydrophilicity/hydrophobicity of the drug being loaded as well on its molecular weight [95,97]. Haney et al. compared some of these loading methods for the loading of catalase, a potent antioxidant, and reported that sonication, extrusion, and saponin treatment provided the highest loading efficiencies when compared to freeze/thaw cycles and passive incubation. It is important to note though that these resulted in an increase in EV size [98]. Additionally, other studies have found that these loading methods compromise the structure and integrity of EV membranes, therefore influencing the therapeutic and immunogenic properties of the drug-loaded EVs [93,97,99].

As mentioned earlier, drug size is also a challenge when loading drugs onto EVs. Large enzymes have been passively loaded onto EVs using saponin-assisted loaded treatment [9,98,99]. Encapsulation of large-scale functional mRNA has also been achieved using cellular nanoporation. Yang et al. transfected various cell sources with plasmid DNAs and stimulated these with electrical stimuli. This enhanced EV production and exosomal mRNA transcripts. However, this requires additional steps during transfection and electrical stimulation, making its industrial translation difficult [9,100].

## 4. Extracellular Vesicle Production and Isolation Methods and Strategies to Enhance Cellular Production of Extracellular Vesicles

**Figure 2:**
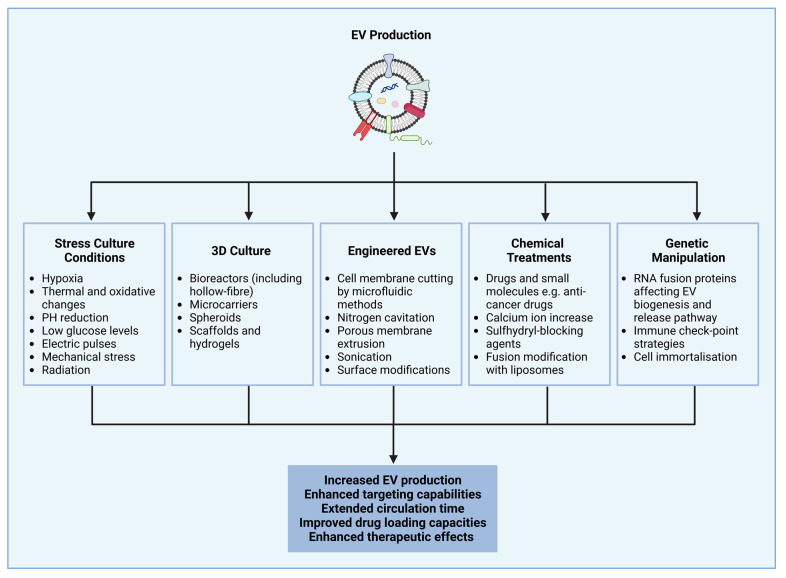
Strategies for enhancing extracellular vesicle production.

When culturing cells in 2D flasks, the surface area of the culture vessel is proportional to cell growth and the quantity of EVs cells produced [101]. Various approaches are currently being explored to increase the yield and quality of exosome secretion, as well as their therapeutic and drug carrier properties, including 3D culture methods, stress inducing factors, genetically manipulating cells, exposing cells to physical stimulation, and chemically treating cells with drugs and other molecules. Additionally, it is also possible to engineer artificial EVs—EV-mimetic nanovesicles—which mimic natural EVs in morphology, size, and function (Figure 2) [102,103].

Although all of the methods suggested above can increase the production of EVs, as well as enhance their properties, the majority of these either induce cellular stress or change the content of the cells secreting them, which will have an impact on the EVs secreted. To overcome this, 3D culture systems have been used for the production of EVs [101].

It is important to mention that despite the effort to enhance EV production, the yields and purities of the isolated EVs will also be dependent on different isolation methods. Different techniques make use of particular EV traits, such as their densities, shapes, sizes, and surface proteins, to achieve isolation. Each of these techniques have different advantages and disadvantages; the most notable are summarised in Table 2.

There are discrepancies between papers on whether UC and dUC are perceived as expensive or not and on whether the isolation produces pure samples. Lai et al., for example, state that UC leads to low-purity samples caused—for example—by protein aggregates [81].

As highlighted in Table 2, microfluidic platforms make it possible to isolate EVs at high purities and yields. Compared to UC, microfluidic chips show higher yields. Zhao et al. developed a microfluidic chip that isolated ~80% of small EVs (below 150 nm) compared to UC, which isolated ~61% [104]. Another advantage of microfluidics over other EV isolation platforms is that it allows for the combined isolation and characterisation of EVs. An example of a useful application of this is liquid biopsies, which offer minimally invasive and real-time molecular profiling of patients [105]. Different isolation methods can be employed within microfluidic platforms, which can be used alone or in combination. These include immunoaffinity, membrane-based filtration, EV trapping on nanowires, acoustic nanofiltration, and deterministic lateral displacement. Detection of EVs can then also be performed using a variety of methods, such as fluorescence and colorimetry detection of EV markers or via assays such as ELISA [106]. In some cases, checking for common exosomal surface markers (CD9, CD63, and CD81), can serve as a disease biomarker diagnosis. For example, the number of exosomes found in human plasma from patients suffering from NSCLC and ovarian cancer was higher than that in healthy patients [107].

In 2014, Kanwar et al. developed the first microfluidic device used for exosome isolation and characterisation. This consisted of a PDMS chip functionalised with antibodies which were able to capture the CD63 exosomal marker. Kanwar et al. used this chip to detect the number of exosomes in serum from healthy and pancreatic cancer patients and showed that there was an increase in the number of exosomes found in pancreatic cancer patient serum [108]. Since then, multiple studies have demonstrated the use of microfluidic platforms to quantify not only EVs generally but also disease-specific EVs by using EV markers specific to each disease. Additionally, it is also possible to use microfluidic platforms to analyse EV miRNAs [109,110,111]. Lim et al., for example, developed a vacuum-driven power-free hydrogel microfluidic chip which was able to detect ERBB2 (a breast cancer marker gene) and a reference gene by amplifying a fluorescent signal via an enzyme-free catalytic hairpin assembly reaction at room temperature. It was tested both in vivo and in vitro and was shown to be effective for the use of liquid biopsies to detect breast cancer [109].

Microfluidics therefore offer a customisable, automatable, scalable, fast, and reliable diagnosis based on EV contents and characteristics. There is still, however, a long process before these can be used in a clinical setting. There is still no “golden standard” for EV isolation methods, and several concepts relating EV biogenesis and roles in cell–cell communication and disease still must be fully understood. In addition, microfluidics can be used to engineer EVs as drug delivery vehicles by encapsulating small molecules, nucleic acids, and proteins into EVs using mainly sonication and electroporation [112].

## 5. An Overview of Different 3D Culture Methods

Traditionally, cells are attached to a flat surface made of glass or plastic, in 2D, as a monolayer. This has become widely used due to its simplicity and convenience and has been an invaluable method of providing insight into a variety of diseases [102]. However, cells cultured in vitro in conventional 2D monolayers have reduced cell–cell and cell–ECM interactions. As such, cells can lose their characteristic morphology and the level of cellular responsiveness is limited. To grow cells in a more natural state, 3D culture methods have been widely used to provide an environment with adequate multidirectional cell–cell and cell–ECM interactions [113]. Additionally, cells cultured in 2D monolayers can lose their phenotype during passaging [114,115]. Since the biogenesis of EVs is not yet fully understood, these changes in cellular organisation could influence our understanding of the characteristics and functions of EVs [41,116].

**Figure 3:**
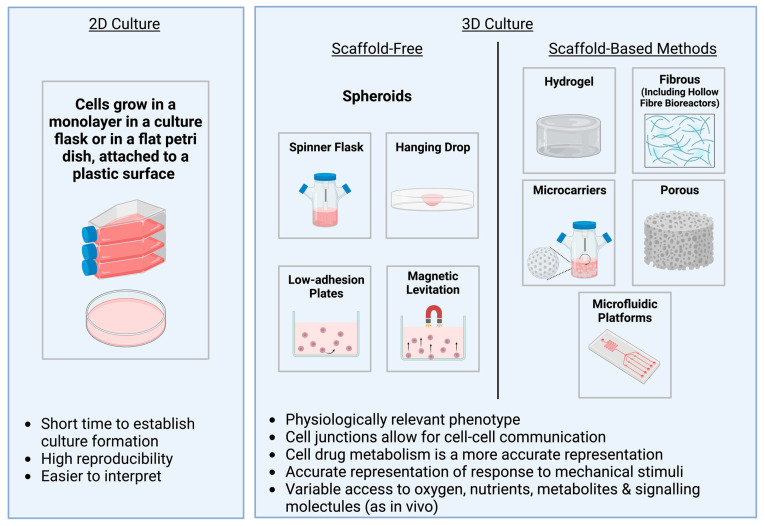
Advantages of 2D and 3D culture methods and the different types of 3D culture approaches.

A wide variety of techniques currently exists to culture cells in 3D. These can be grouped into two main categories: scaffold-based methods and scaffold-free methods (Figure 3). Scaffold-free methods involve mostly spheroids, which are formed without attachment to a scaffold; therefore, cell–cell adhesion is required for their formation [113]. Scaffold-based methods, on the other hand, use exogenous materials to provide a substitute for the tissue’s native ECM. Modification of the chemical and physical properties of these allows mimicry of the microenvironment found in specific tissues and provide greater structural integrity for larger tissue engineered constructs [117]. Scaffold-based methods include hydrogels, fibrous and porous scaffolds, and microcarriers amongst others. The wide range of techniques available make it possible to choose from a range of biocompatible materials which give rise to different structures and architectures and mechanical and degradation properties, which can be tailored for different applications [118].

## 6. Spheroids

Spheroid culture consists of cell aggregates (usually ranging in diameter from 50–1000 µm) caused by the self-assembly of cells when cultured in suspension [117]. Spheroid 3D culture methods allow for cell–cell communication, better mimicking of in vivo environments, and retention of the intrinsic phenotypic properties of cells [119]. Due to their simplicity, low cost, and reproducibility, spheroid cultures allow for automated production of hundreds to thousands of spheroids within 24 h, which is advantageous for high-throughput drug toxicity screening and other applications [117].

For spheroids in the size range of hundreds of micrometres, it becomes difficult to transport oxygen and nutrients to the core and to dispose of metabolic waste products via simple diffusion [114]. This causes concentration gradients in which the inner cells have less access to nutrients and suffer accumulation of toxic waste products, causing necrosis in the centre of spheroids, which leads to self-disassembly. The sizes of spheroids for which this occurs are dependent on the cell type [119]. Necrosis is characteristic of tumour environments and therefore makes spheroids attractive for studying cancer-associated pathways and the effects of anticancer drugs [120].

Although spheroid 3D cultured cells are not provided with an external ECM, spheroid cells are able to secrete ECM proteins. ECM protein secretion in spheroid culture is dependent on the size of the spheroid and cell type [117]. For example, when staining human glioma and human thyroid cancer cell lines grown in spheroid culture for fibronectin, laminin, and collagen (ECM proteins), it was found that both spheroid types presented ECM proteins [121]. In contrast, spheroid culture of fibroblasts leads to nemosis, caused by cell–cell contact inhibition and the need for a sufficiently rigid mechanical environment, which eventually causes cell death. The major signalling pathway associated with this is the Hippo signalling pathway, which leads to an inhibition of the transcriptional effectors YAP and TAZ, which have essential roles in cell proliferation, migration, and survival [122,123].

Over the past decade, a variety of methods have been developed in order to achieve spheroid culturing techniques. The most commonly used methods of obtaining spheroid culture are the hanging drop method, using low-adhesion plates, stirring cells in a spinner flask, and magnetic levitation. The hanging drop method consists of placing culture medium on petri dish lids. Cells then accumulate at the tip of a drop and aggregate spontaneously, leading to the formation of spheroids. However, this method does not allow for change in culture medium, limiting the duration of cell culture, making scaling-up processes not feasible [124]. Alternatively, cells can be placed in a low-adhesion plate so that the cells cannot attach to the substrate and instead aggregate with each other, forming spheroids [125]. Another method of forming spheroids is placing cells in a spinner flask. This will agitate the cells to form spheroids, allowing for larger scale production [126]. Magnetic levitation involves labelling cells with magnetic nanoparticles and subsequently applying an external magnetic field, encouraging cells to self-assemble into multicellular spheroids. This technique overcomes the limitation of core spheroid necrosis and includes other advantages, such as the ability to image and track the cells [127].

Spheroid culture has been used successfully to culture cells and isolate EVs. Faruqu et al., for example, isolated exosomes from umbilical-cord-derived MSC (UC-MSC) spheroids. These exosomes caused increased migration and proliferation of murine fibroblasts in vitro, therefore having the potential of accelerating wound healing [128]. Many studies have also shown that MSC cultured in 3D spheroid conditions produced more EVs compared to when being cultured in 2D methods. This was shown by Kim et al., who demonstrated that when seeding MSC at the same density, a higher number of exosomes was produced from spheroid culture vs. traditional culture methods [129]. Xie et al. isolated MVs from MSC cultured in 2D and in spheroid conditions and found that, compared to 2D-MSC-MVs, 3D-MSC-MVs significantly decreased the chemotactic index of CD14+ cells and experienced an increased capability to induce signal factors as well as to improve immunomodulating activities, suggesting that these might be a better regenerative therapy for retinal degenerative diseases [130]. Miceli et al. compared the immunosuppressive and angiogenic properties of MSC-derived exosomes between 2D and 3D spheroid culture and found that there was no significant difference between 2D- and 3D-spheroid-culture-derived exosomes inducing capillary-like formation and endothelial cell migration. However, there was an increased capillary maturation and inhibition of peripheral blood mononuclear cells (PBMC) in the presence of 3D-spheroid-culture-derived condition medium (CM) compared to 2D-culture-derived CM and 2D-culture-derived exosomes [131].

Similar results have also been obtained with different cell types. A study by Hu et al. showed that exosomes secreted by spheroid culture of dermal papilla (DP) cells had a more profound therapeutic effect. Their group investigated the effect of these in hair growth and showed that miR-218-5p was notably up-regulated in exosomes from DP cells grown in spheroid culture when compared to 2D methods. This enhanced the expression of β-catenin and down-regulated secreted frizzled-related protein (SFRP2), which positively regulated hair follicle growth and maintained the anagen phase of the hair cycle [132].

The same research group (Hu et al.) also compared the effects of human dermal fibroblasts (HDF)-derived 2D and 3D spheroid exosomes on skin photoaging. They demonstrated that 3D spheroid-culture-derived exosomes caused increased procollagen type I expression and a significant decrease in matrix metalloproteinases 1 (MMP-1) expression as well as a higher levels of dermal collagen deposition. This indicated that exosomes derived from HDF spheroid 3D culture have anti-skin-aging properties and the potential to prevent and treat cutaneous ageing [133].

Tu et al., used exosomes to demonstrate that cancer cell spheroids are more physiologically relevant to native tumour tissue than 2D cultured cells. Exosomes were isolated from a pancreatic cancer cell line (PANC-1) grown in 3D spheroids and in 2D culture flasks. Spheroid 3D culture methods yielded a higher number of exosomes which contained miRNA and *Glypican-1* (GPC-1), more closely resembling the progression of pancreatic cancer [134]. Naseri et al., cultured cancer stem cells (CSC) in spheroid form and tested the exosomes secreted by these and their impact on dendritic cells (DC) maturation in order to better understand the therapeutic impact of DC against CSC. DC spheroid exosomes increased the ratio of interleukin-12 (IL-12) to interleukin-10 (IL-10) supernatants of mature DC, favouring immunostimulation and promoting T cell proliferation. T cells treated with these exosomes disrupted spheroid structure presenting an alternative immunotherapy strategy to reduce cancer spread [135]. Liu et al. also analysed the effect of exosomes from CSC and found that breast CSC spheroid exosomes had a significant impact in breast cancer cell glycolysis by miR-1252-5p/PFKB2 [136].

In 2020, a series of studies were conducted by Dinh et al. in which lung spheroid-cell-derived secretome (LSC-Sec), lung spheroid-cell-derived exosomes (LSC-exo), and MSC-derived secretome and exosomes were used to treat different models of lung injury and fibrosis. This study revealed that all secretome- and exosome-treated groups had a therapeutic benefit: decreased fibrosis and reduced lung apoptosis. However, only LSC-Sec and LSC-exo showed a more notable effect and decreased collagen deposition [137].

Additionally, spheroids have also been used to mimic a more representative form of the in vivo microenvironment to test the effect of exosomes. Choi et al., compared the effects of exosomes isolated from the CM of head and neck squamous cell carcinoma (HNSCC) and primary-cancer-associated fibroblasts (CAF) coculture from 2D conditions and 3D spheroids on angiogenesis in HSNCC. Their results showed that exosomes derived from 3D spheroid culture upregulated angiogenesis-related genes in HNSCC 3D spheroid culture when compared to 2D. Their results also showed that vasculature was significantly increased in mouse tumour xenograft models derived from 3D spheroids of HNSCC cells with primary CAF when compared to tumours derived from cells cultured in monolayers. This makes the tumour model derived from spheroids a more reliable model for studying the function of exosomes and for obtaining chemotherapy drug screening results [138].

Verdera et al. performed a set of experiments to characterise the cellular mechanisms involved in EV uptake. In their work, they showed that the uptake of EVs in 3D spheroids was restricted to the outer few cell layers in contrast to the 2D monolayer, where all cells had taken up EVs within 4 h. The 3D spheroid model mimics more closely in vivo cellular organisation, suggesting that spheroids might be best to test EV internalisation and therefore EV therapeutics and drug delivery [139].

Organoid systems also take advantage of the self-organising capabilities of cells to recreate in vitro miniaturised and simplified model systems of organs. Organoids have also been used for the research of EVs. The current review does not cover the production of EVs from scaffold-free multicellular organoids, which has been reviewed elsewhere [140].

## 7. Hydrogels

Hydrogels are 3D hydrophilic polymers that have attracted attention due to their low cost, ease of use and range of materials they comprise of [141]. Hydrogels can be made of either natural polymers (e.g., gelatin, fibrin, alginate, agarose) or synthetic polymers (e.g., polyethylene glycol (PEG), poly(acrylic acid) (PAA), polyethylene oxide (PEO), polyvinyl alcohol (PVA)). When cross-linked through either covalent or non-covalent bonds, hydrophilic polymers absorb water (up to ~95% of hydrogel content) [142] and swell without dissolving. Higher cross-linking results in lower swelling [143]. In the swollen state, they are often soft and elastic, making them ideal for mimicking soft tissue. Additionally, hydrogels allow free diffusion of soluble factors such as cytokines and growth factors needed for cell growth [119]. Control of hydrogels’ swelling can be used to tailor their mechanical properties, such as rigidity, making it possible to match different tissue stiffness [144]. Their rate of degradation can also be tailored, allowing sustained release of encapsulated materials [114,141,145].

Porosity can also be introduced into hydrogels either through cross-linking or phase separation during synthesis. The pore size, distribution, and interconnections all have an impact on the mechanical properties of the hydrogel. However, in hydrogels, it can be difficult to obtain homogenous pore distributions and sizes, which may have an impact on the surface area for cell growth [146]. Pore sizes are important in enabling adequate tissue growth and are dependent on the specific type of cell. For example, hydrogels with pore sizes of 25–500 µm are required for cartilage growth, ~166 µm for vascular network formation, and 50–200 μm for the growth of smooth muscle cells [147,148].

The relatively low mechanical strength of hydrogels limits their application in tissue engineering to mainly soft tissues, such as skin [146,149,150]. Nonetheless, hydrogels have been proven to be useful in EV research. Using stiffness tuneable ECM hydrogel scaffolds, Patwardhan et al. reported that stiff ECMs promote exosome secretion in a YAP/TAZ-pathway-dependent manner. To do this, MCF-7 and MDA-MB-231 cells (epithelial human breast cancer cell lines) were grown in polyacrylamide gels (PA), which were coated with different amounts of collagen to obtain hydrogels with different stiffness. This study showed that hydrogels can be used to increase exosome production as well as to provide a better method of understanding breast cancer [151].

Other studies have used hydrogels to grow cells and isolate the EVs secreted. Thippabhotla et al. used peptide hydrogels to grow HeLa cells and compared the EVs secreted by these to those secreted by HeLa cells grown in 2D monolayers. Their study showed that hydrogel 3D-culture-derived EV small RNAs were different from their originator cells’ RNA profile, possibly indicating a specific sorting process. Additionally, they showed that hydrogel-derived 3D EV small RNAs exhibited a much higher similarity to in-vivo-circulating EVs derived from cervical cancer patient plasma. However, DNA sequencing analysis revealed that there was no difference in the genomic information carried by EVs based on the cell growth conditions. Their work shows that EVs could be used as molecular indicators in models used to study the effect of drugs or treatments and that hydrogel scaffolds are a more physiologically representative models to grow cells compared to 2D monolayers [25].

Han et al. used gelatin methacryloyl (GelMA) hydrogels to grow MSC and then compared the exosomes secreted by these with exosomes secreted by MSC cultured in 2D monolayers. They found that exosomes derived from hydrogel 3D cultures showed better neuroprotective effects compared to exosomes derived from 2D cultures. Additionally, exosomes derived from hydrogel 3D cultures contained a higher component of neuroprotective-related proteins and miRNAs that regulate the transcription of cellular immune responses genes. Furthermore, exosomes derived from hydrogel 3D cultures contained a higher amount of anti-inflammatory and anti-apoptotic factors which could be beneficial for the treatment of spinal cord injury (SCI). The same hydrogel was investigated for the delivery of exosomes derived from hydrogel 3D cultures to a SCI lesion site. This significantly improved their retention at the site and achieved a more sustained release when compared to repeated local injection of exosomes [152].

Other research groups have similarly used hydrogels for sustained delivery of EVs. These hydrogels tend to have low viscosity fluid with shear-thinning properties, improving retention at the site of injection [153]. There are three common approaches by which EV encapsulation into hydrogels is achieved. The first involves combining exosomes with polymers followed by the addition of crosslinkers inducing gelation. The second approach is commonly known as the “breathing technique” and involves soaking the swollen hydrogel in a solution containing exosomes, which will enter the porous hydrogel network. The third approach involves mixing the exosomes with both the polymer in solution and the crosslinkers simultaneously, causing in situ gelation [141,154]. Chen et al., for example, used an injectable hyaluronic acid (HA) hydrogel to deliver endothelial progenitor cell (EPC)-derived EVs into the ischaemic myocardium. This study showed increased therapeutic efficiency and efficacy towards cardiomyocyte recovery [155]. The most commonly used methods for EV delivery using hydrogel-based systems are summarised in Table 3.

## 8. Hard Porous and Fibrous Scaffolds

Hard scaffolds consisting of 3D structures with interconnected pores or fibres are also widely used to provide a physical support for cell growth and attachment. Compared to hydrogel scaffolds, they have higher mechanical strength, stiffness, and wear resistance [172]. As with hydrogels, hard scaffolds can be made of natural or synthetic polymers. Natural polymers often have inherent bioactivity and may be more biocompatible. Synthetic polymers, on the other hand, are more versatile, have increased reproducibility, and are easier to process, allowing for more control over structural features [173]. Pore and fibre structures, such as their interconnection and arrangement (orientated or random), determine the scaffold’s surface area and mechanical properties, which may influence cell phenotype, vascularisation, and ECM deposition [141]. These will also influence cellular penetration and migration to the scaffold’s interior and access to nutrients and oxygen [114].

Currently, the most common applications of hard scaffolds are to recreate the physical and structural environment of tissues, such as bone, cartilage, vascular, ligament, and skeletal muscle, as well as applications in preclinical in vitro testing of spheroids, tumoroids, and organoids [174]. It is often important that the scaffold’s mechanical properties mirror those of the target tissue and enable stress transfer and load bearing. The degradation profile should also be chosen carefully so that the scaffold provides the necessary support during tissue formation [115].

Traditional techniques for scaffold fabrication include solvent casting and particulate leaching, gas foaming, thermally induced phase separation (TIPS), and freeze-drying. These techniques have poor reproducibility and poor control over defined pore and fibre structures [118]. Advanced scaffold fabrication techniques have been developed in order to achieve better control over the aforementioned parameters. These include fused deposition modelling, stereolithography, 3D printing, the integration of computer aided design (CAD) software, electrospinning, and rapid prototyping [175].

Electrospinning has the capability of controlling the diameter of the nanoscale thin fibres, obtaining a homogenous fibrous scaffold. Electrospun scaffolds have been used for cell culture and EV collection. Kadir et al. generated electrospun fibre sheet scaffolds to grow MSC. Their results compared the CM generated from MSC grown on the fibres to the CM of MSC grown in traditional culture plates. CM from scaffold 3D culture showed enhanced migration and proliferation of MSC and chondrocytes as well as reduced inflammation. They also investigated different fibre alignments and found that these also had an effect on the properties of the CM [176].

Hu et al. showed similar results. Sericin-deprived spun silk from comber (a waste material derived from cotton manufacturing) was bonded via mechanical hydroentanglement in order to achieve web formation. Adult HDF were then cultured on the silk fibroin woven scaffolds. Compared to the exosomes from HDF grown in standard 2D flasks, 3D exosomes carried higher amounts of angiogenic growth factors, and they also induced human dermal microvascular endothelial cells (hDMVEC) to form a higher number of tubes in vitro [177].

Three-dimensional printing can also influence scaffold pore configuration, and therefore, scaffolds can be tailored to increase EV production and/or enhance their clinical potential. Gao et al. cultured BM-MSC on 3D-printed hydroxyapatite scaffolds and investigated the effect of the isolated exosomes on cell proliferation, migration, tube formation, and angiogenesis in vivo. These exhibited stronger effects than exosomes isolated from the same cell line cultured using traditional culture methods [178]. Man et al. also showed that culturing cells in 3D-printed scaffolds enhanced the therapeutic potential of the isolated EVs. In their study, osteoblasts were cultured on 3D-printed titanium scaffolds coated with hydroxyapatite with different pore sizes and shapes, and the effect this had on EV secretion yield and on the EV capacity to induce osteogenic differentiation was studied. Triangular pore geometry and coating increased bone mineralisation and EV production (2.2-fold and 4.5-fold), demonstrating that it is possible to harness bone-mimetic culture platforms, enhancing the production of pro-regenerative EVs [179]. Liu et al. used 3D printing for exosome delivery. They pretreated a 3D printed collagen/silk fibroin scaffold with hypoxia-pretreated human UC-MSC (hUC-MSC)-derived exosomes and implanted this into a traumatic brain injury (TBI) beagle model. Their study revealed that the implanted exosome scaffold inhibited nerve cell apoptosis and inflammation, enhancing the motor functional recovery of TBI [180]. Chen et al. used coaxial 3D printing to print a meter-long hollow hydrogel microfibre, which allowed for the expansion of MSC. Their platform enriched particles ~1009-fold when compared to 2D culture, and EVs secreted from scaffold 3D cultures conserved their pro-angiogenic properties [181].

It is also possible to incorporate EVs into 3D-printable bio-ink. Born et al. incorporated MSC-derived EVs into GelMA hydrogel bio-ink and demonstrated that increasing the concentration of a crosslinker during gelation can improve the sustained release of the pro-angiogenic EVs [182]. Chen et al., used a bio-ink composed of MSC-derived exosomes, decellularized cartilage ECM, and GelMA hydrogel to 3D print a biocompatible scaffold which was implanted on a rabbit model with an osteochondral defect. Their results showed that the ECM/GelMA/exosome scaffold was able to facilitate cartilage regeneration in vivo [183].

Pavia et al. used TIPS to prepare poly-L-lactic acid (PLLA) scaffolds and changed the processing parameters to compare two different morphologies. Their results showed that astrocytes were able to secrete exosomes when cultured on both scaffold morphologies [184]. Another example of this was shown by Zhang et al., who demonstrated that culturing hBM-MSC in 3D collagen scaffolds had a stronger outcome in enhancing spatial learning compared to exosomes obtained from hBM-MSC cultured in 2D platforms when injected in a TBI rat model [185]. Scaffolds and grafts have been used widely in bone regeneration by fabrication of hybrid biomaterials with multipotential cells and their released factors [139]. Table 4 summarises exosome-integrated bone engineering scaffolds strategies used to date.

Scaffolds have also been used for the sustained delivery of EVs as drug delivery vehicles. Zhang et al. encapsulated paclitaxel (PTX) onto MSC-derived exosomes and seeded these onto a collagen scaffold. This allowed for the controlled release of exosome encapsulated PTX and showed superior performance for motor functional recovery when implanted on SCI rat models. [186]

**Table 4:** Scaffold–exosome approaches for bone regeneration.

Type of Scaffold	Cell Type	Applications	References
Type I collagen and fibronectin matrix proteins	MSC	Augment performance of lineage specific differentiation of naïve MSCs in bone transplantation	[187]
Porous β-tricalcium phosphate	iPSC and MSC	Increased angiogenesis and osteogenesis	[188,189,190]
PCL	MSC and chondrogenic ATDC5 cells	Enhanced osteogenic differentiation	[191,192,193]
Mineral-doped PLA porous	hAD-MSC	Enhanced osteogenic MSC differentiation	[194]
Biodegradable PLGA	hAD-MSC	Enhanced osteogenic differentiation and enhanced mineralisation by endogenous cell recruitment	[195,196]
Tannic-acid-modified sulfonated SPEEK	BM-MSC	Enhanced osteoimmunomodulation by promotion of macrophage polarisation	[197]
3D-printed porous Ti alloy (Ti6Al4V)	SC	Improved efficacy of Ti alloy scaffolds in bone repair	[198]
Calcium sulphate hydroxyapatite nanocement	MSC	Enhanced bone mineralisation	[199]

Abbreviations: iPSC, induced pluripotent stem cells; PCL, polycaprolactone; hAD-MSC, human AD-MSC; PLA, poly(L-lactide) acid; PLGA, poly(lactic-co-glycolic acid); SPEEK, polyetheretherketone; Ti, titanium; SC, Schwann cells.

## 9. Microcarriers

The use of microcarriers to culture cells in suspension has been applied for the production of vaccines and pharmaceuticals, as a delivery method, and to expand cell populations. Microcarriers were successfully introduced in 1967, and there is now a large number of commercially available microcarriers comprising different sizes, materials and surface topographies [111]. Microcarriers are spherical beads with sizes typically in the range of 100–300 µm. They provide a substrate that enables attachment of adherent cells in bioreactors that can be maintained in suspension using agitation [200]. Several studies have demonstrated that the process of agitation may mechanically stimulate the cultured cells resulting in a higher EV yield [97]. Patel et al., for example, demonstrated that applying a low shear rate on human dermal microvascular endothelial cells (HDMEC) cultured on a perfusion bioreactor system increased EV secretion 100-fold [201]. Additionally, agitation creates a homogenous environment that allows cells to obtain uniform exposure to oxygen and nutrients [202]. However, agitation must be carefully controlled to avoid cell damage and cellular shedding of the microcarriers. To grow cells in a bioreactor, two strategies are typically implemented. The first consists of spheroid formation, as described in Section 6, and the second one consists of using suspension culture systems such as microcarriers, to which adherent cells can attach and grow [203].

Many mammalian cells are anchorage-dependent, and therefore, cell attachment to the microcarriers’ surface is a crucial parameter influencing metabolic activity, growth, and culture productivity [204,205]. The process of cell attachment results from interaction between the cell’s transmembrane surface receptors, such as integrins, and proteins expressed on the surface of the microcarrier. Under normal culture conditions, attachment proteins such as vitronectin and fibronectin found in the serum added to culture medium coat the surface of the microcarrier [204,206]. However, certain cells, such as fibroblasts, secrete high amounts of fibronectin and therefore do not require exogenous proteins for attachment [207]. Additionally, all vertebrate cells possess unevenly distributed negative charge on their surface and will therefore attach to positively charged microcarriers. Non-charged and negatively charged microcarriers are also available; these tend to be coated with ECM proteins to facilitate cell attachment [208]. Microcarriers can be hydrophilic or hydrophobic. Hydrophilic surfaces allow for better protein absorption and therefore lead to a higher cell attachment [204].

**Figure 4:**
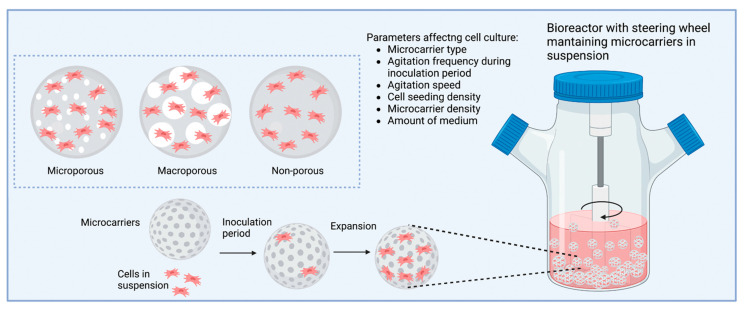
Schematic of cell attachment onto microcarriers and the different parameters affecting their culture.

Microcarriers can be divided into three main categories: non-porous, micro-porous, and macro-porous microcarriers (Figure 4). In solid and micro-porous microcarriers (pores are ≤10 µm), cells grow as monolayers on their surfaces. However, in macro-porous microcarriers (pores are 10–50 µm), cells can potentially migrate and proliferate within the porous matrix as well as on the microcarrier’s surface, providing a larger surface area for growth. Furthermore, cells found within the pores of the microcarriers are protected from exposure to mechanical damage that may occur during agitation [209,210]. Macro-porous microcarriers are also suitable for immobilising non-adherent cell types. The microcarrier’s shape (such as spherical, disc, or cylindrical) also influences cell attachment. For example, Schmidt et al. demonstrated that MSC have a lower proliferative index with more curved microcarrier surfaces due to increased cell exposure to high shear stresses in bioreactors [211].

The material a microcarrier is made of is very important since it can affect the stiffness, hydrophobic/hydrophilic properties, permeability (important for the transport of oxygen and nutrients), and topography of the microcarrier [208]. The materials used to produce microcarriers can be divided into two categories: synthetic and natural polymers. Synthetic polymers are often biocompatible and biodegradable, including poly(lactic-co-glycolic acid) (PLGA), polylactic acid (PLA), poly(ε-caprolactone) (PCL), polystyrene, and dextran. Natural polymers include cellulose, gelatine, collagen, alginate, and chitosan [111,198,200]. Additionally, multiple culture parameters, such as the cell and microcarrier seeding density, can also impact cell attachment and growth [208].

Multiple research groups have successfully obtained EVs from cells attached to microcarriers (Table 5). In 2018, Čebatariūnienė et al. grew periodontal ligament stem cells (PLSC) on gelatin-coated alginate microcarriers using a bioreactor. NFkB reporter assays demonstrated that the EVs secreted by these stem cells suppressed basal and lipopolysaccharide (LPS) induced activity of NFκB activity, suggesting that these could potentially be used for targeting chronic inflammation during periodontitis [212]. 

Additionally, multiple papers have focused on looking at the implication of using microcarriers to culture cells to produce higher yields of EVs (Table 5). Fuzeta et al. used a serum-free/xeno-free microcarrier-based culture system implemented in a vertical wheel bioreactor in order to achieve the scalable production of exosomes derived from MSC obtained from different tissue sources. This allowed production of exosomes at a higher concentrations, productivities and purities [202]. 

Xu et al. demonstrated that the exosomes derived from microcarrier-based 3D-cultured hUC-MSC had the potential to inhibit silica-induced pulmonary fibrosis in vivo and improved lung function as a result. These results were confirmed in vitro, where it was shown that the exosomes reduced collagen deposition in NIH-3T3 cells [213].

Haraszti et al. cultivated hUC-MSC on spherical support matrix beads (Star-Plus Microcarriers) stirred in a spinner flask using serum-free medium and compared the exosomes secreted by these cells to those secreted by hUC-MSC grown in 2D culture flasks. They also compared two different exosome isolation methods: UC and tangential flow filtration (TFF). This research group showed that microcarrier-based 3D culture yielded up to 20-fold more exosomes compared to 2D culture. Additionally, their findings also reported that microcarrier 3D-TFF exosomes were seven times more potent in small interfering RNA (siRNA) transfer to neurons when compared to 2D exosomes [214].

Additionally, Jalilian et al. also successfully demonstrated that EVs derived from cells isolated in microcarriers have a more potent clinical potential. Jalilian et al. cultured BM-MSC in 2D monolayer and in a vertical wheel bioreactor using Corning’s Low Concentration Synthemax II microcarriers and isolated the secreted EVs in order to investigate the potential differences in neuroregenerative properties of the EVs generated in 2D and micro-carrier-based 3D culture conditions. NTA showed that there was a 24-fold increase in EV concentration for microcarrier-based 3D culture. Additionally, a notable shift toward a more heterogenous phenotype was observed in microcarrier-based 3D-culture-derived EVs when compared to 2D-culture-derived EVs. Both 2D and microcarrier-based 3D-culture-derived EVs induced neurite growth, but microcarrier-based 3D-culture-derived EVs showed a significant increase in neurite length in trigeminal ganglia (TG) neurons when compared to 2D EVs in vitro [215].

In 2022, Cytodex microcarriers were used in order to grow human synovial fluid MSC (hSF-MSC) in a perfusion bioreactor. Compared to 2D culture, hSF-MSC prolifer-ated more quickly from 6 days onwards. After 14 days, exosomes were isolated using UC, and the samples were subsequently analysed. Both nanoparticle tracking analysis (NTA) and western blots revealed a higher exosomal concentration for microcarrier-based 3D culture samples. Exosomes derived from the microcarrier-based 3D culture system also delivered miRNAs into cells, suggesting that culturing MSC is a microcarrier-based culture system does not interfere with exosome function. Microbial and endotoxin detection results suggested that the protocol can produce exosomes which are biologically safe and promising for osteoarthritis therapy [216].

In 2015, a study revealed that exosomes derived from the microcarrier-based 3D culture of stem cells derived from dental pulp of human exfoliated deciduous teeth (SHED) supressed 6-hydroxy-dopamine (6-OHDAe), which induced apoptosis in dopaminergic neurons. This was not observed from exosomes derived from SHED grown in standard 2D culture flasks and could be a hallmark discovery for the treatment of treatment of Parkinson’s disease [217].

Additionally, microcarriers have also been used to encapsulate EVs as diagnostics tools. Hou et al. used hydrogel microcarriers to identify tumour-derived exosomes by immobilising microcarriers with HER2 aptamers, which are transmembrane proteins expressed in HepG2 exosomes [218].

## 10. Hollow Fibre Bioreactor

**Figure 5:**
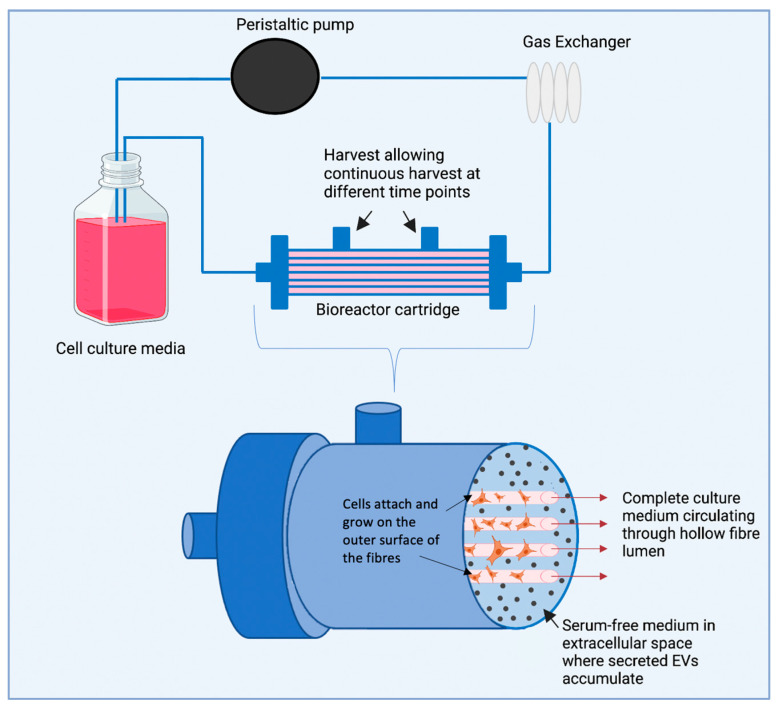
Schematic diagram of a hollow fibre bioreactor. Adapted with permission from I. K. Yan, N. Shukla, D. A. Borrelli and T. Patel, Methods in Molecular Biology; published by Springer Nature, 2018 [222].

A hollow fibre bioreactor is a type of bioreactor used for culturing cells in 3D that can also be used to obtain high concentrations of EVs [219]. The device consists of many small, semi-permeable hollow fibres bundled together in a tubular shell. In this way, cells can be seeded on the outside of the hollow fibres and media can be delivered through the fibre lumen. The system comprises a large surface area, which allows for a high seeding density and cell growth under a homogenous controlled culture environment. Using this system over microcarriers has the advantage of fibres protecting cells from shear stresses and allows for culture medium to circulate through the fibres where the cells are attached, while serum-free media can be applied in the extra capillary space (where EVs are secreted for isolation), preventing cross-contamination from other protein aggregates (Figure 5) [220,221,222].

In 2020, a study by Cao et al., evaluated the effect of culturing hUC-MSC in a hollow fibre bioreactor on the secreted exosomes. This culturing method yielded up to a 19.4-fold increase (as shown by protein assays) in exosome concentration when compared to traditional 2D culture. Tubular epithelial cells (TEC) were used to compare the therapeutic effect of exosomes derived from 2D cultures with exosomes derived from hollow fibre bioreactor 3D cultures. The results showed that exosomes derived from 3D cultures were more easily taken up by TEC and had a higher ability to reduce cisplatin-induced inflammation and to improve the viability of TEC. Furthermore, after injecting AKI mice models with exosomes derived from 2D and hollow fibre bioreactor 3D cultures, it was found that exosomes derived from hollow fibre bioreactor 3D cultures were more effective at alleviating AKI, which was shown by improved renal function, attenuated pathological changes of renal tubules, reduced inflammatory factors, and repressed T cell and macrophage infiltration [28].

Yan et al. investigated the use of hollow fibre bioreactors in producing exosomes for cartilage repair. UC-MSC were grown in a hollow fibre bioreactor, and these were found to yield 5.7 times more exosomes than 2D culture methods when compared using NTA. Additionally, results indicated that exosomes derived from 2D and hollow fibre bioreactor 3D cultures stimulated chondrocyte proliferation, migration, and matrix synthesis and also inhibited apoptosis, with exosomes derived from hollow fibre 3D cultures exerting a stronger effect. The exosomal therapeutic effects were then assessed by injecting exosomes derived from 2D and 3D cultures in cartilage defect areas. Exosomes derived from hollow fibre bioreactor 3D cultures showed a superior therapeutic effect [223].

Waston et al., also used a hollow fibre bioreactor for the efficient production of EVs. In their work, HEK293 cells expressing hetIL-15 were grown on a hollow fibre culture system and compared to those grown in conventional culture methods. They showed that this method yielded ~40-fold more EVs per mL while retaining their surface properties. Additionally, biophysical, and comparative proteomics suggested a more diverse population of EVs in the bioreactor preparations, while serum protein contaminants were detectable only in conventional culture EV preparations [224].

## 11. Conclusions

In recent years, there has been an increasing number of preclinical and clinical studies that have confirmed the therapeutic impact, drug delivery capacity and diagnostic potential of EVs. EV yield is a major limitation to the clinical translation of EV technology and as a result a wide range of studies have focused on different strategies to increase EV production.

Currently, to obtain EVs, cells are grown in 2D flaks, for which the surface area of the culture vessel is a limitation to the amount of EVs that can be obtained. Growing cells in monolayers results in reduced cell–cell interactions, influencing EV components and functions, which can hinder EV research for medical applications. The use of 3D culture methods has been shown to allow the expansion of a large number of cells, reducing cost and time required for culture. Additionally, 3D-cultured cells retain their phenotype and several studies have shown that 3D-culture-derived EVs have more enhanced clinical use. Furthermore, 3D culture methods have also been used for the delivery of EVs, allowing for a more sustained release and greater retention at the target site.

Different 3D culture methods present unique sets of advantages and disadvantages and have been used for different applications in EV research. Spheroid culture is cost efficient and reproducible; however, the transport of oxygen and nutrients to the inner cells in large spheroids is difficult, leading to cell death and therefore reducing the amount of EVs that can be obtained from spheroid 3D cell culture. Spheroid culture has been used successfully to culture cells and isolate in-vivo-like EVs. Additionally, the 3D spheroid model mimics more closely in vivo cellular organisation and therefore has been used to test EV therapeutics and drug delivery. Hydrogels offer low cost and ease of use and are comprised of a range of materials that are readily available. However, it is difficult to control pore size and distributions. This and hydrogels’ low stiffness limit their application to mainly soft tissues. Hydrogels have, as spheroids, been used to grow cells and isolate EVs with stronger therapeutic effects both in vivo and for the sustained delivery of EVs. Hard and porous scaffolds have also been used to isolate EVs from cells grown in a microenvironment more closely resembling in vivo and for the delivery of EV’s to certain tissue, achieving an enhanced therapeutic effect. Compared to hydrogel scaffolds, they have higher mechanical strength, stiffness, and wear resistance. They also have increased reproducibility and are easier to process, allowing for more control over structural features. However, producing scaffolds with controlled structures is expensive. Microcarriers, on the other hand, are widely commercially available, making them more affordable and offering a range of different sizes, materials, and surface topographies. When growing cells using microcarriers, these are steered in a bioreactor, and shear stress could potentially damage these cells. Multiple research groups have successfully used microcarriers to grow cells to obtain EVs with enhanced therapeutic effects and to increase EV production yield. Hollow fibre bioreactors offer a larger surface area and have an advantage over microcarriers, as the fibres protect cells from shear stress. They also allow for the growth of cells in a different space from where EVs are secreted, preventing cross-contamination from other EVs found in the cell culture medium.

Three-dimensional delivery and cell growth platforms can be tailored depending on the application and scale required. This review article summarises the different methods used for EV production and delivery. Careful consideration should be taken by the user before choosing the 3D methods for EV studies to obtain the desired clinical application.

## Figures and Tables

**Table 1:** The different types of extracellular vesicles and their defining characteristics.: miRNA, microRNA; MVs, microvesicles.

Vesicle	Size	Origin	Contents	Markers	References
Exomeres	≤50 nm	Unknown	DNA, RNA, miRNAs and lipids	Unclear—More studies are required	[7,30]
Exosomes	30–200 nm *	Intra-luminal budding into MVBs and release by MVB fusion with cell membrane	Membrane proteins, different RNA species, lipids and DNA	Tetraspanins, heat-shock proteins, integrins, TSG101, flotillin, MFGE8 and ESCRT components. They include cell-type-specific proteins	[1,3,31,32]
MVs	10–1000 nm	Outward budding or blebbing of cell plasma membrane	Membrane proteins, different RNA species, lipids, and DNA	Annexin A1 (on MVs that shed directly from the plasma membrane), integrins, selectins, and CD40 ligand, phosphatidylserine	[3,29,31,32,33]
Apoptotic bodies	1–5 µm	Outward blebbing of apoptotic cell plasma membrane	Nuclear fractions, cell organelles and degraded proteins	Annexin V and high amounts of phosphatidylserine	[3,31,32]

* The size range of exosomes reported in published literature differs. For example, Sun et al. reported a size of range of 30–120 nm [34], Jeppesen et al. reported 40–150 nm [33], and diameters of up to ~200 nm have been measured using cryogenic transmission electron microscopy (cryo-TEM) [35]. Inconsistency in methodology used for the collection, isolation, and analysis of exosomes, as well as size variation between exosomes from different cell types, is likely to have contributed to these differences.

**Table 2:** Different methods of isolating EVs and their associated advantages and disadvantages.

Method	Principles & Materials	Advantages	Disadvantages	References
Differential Ultracentrifugation (dUC)	Physical—Components with imparity of size and density possess various sediment speeds	Gold standard Low cost Pure samples Suitable for large sample volumes	Time-consuming Low yield Repeated and high-speed steps might damage EVs	[19,41,44,91]
Density Gradient Centrifugation	Physical—Components with imparity of size and density possess various sediment speeds	Higher purity than dUC Maintains EVs intact	Time-consuming Low yield	[19,68]
Ultrafiltration (UF)	Physical—Filters particles with various sizes and molecular weights	Quick and simple High yield	Low purity EV deformation	[19,41,68,81,91]
Precipitation	Physical/Chemical—High hydrophilic polymers influence the solubility of EVs	High yield Easy Low cost Concentrates diluted samples	Low purity Potential contaminants (co-purifying protein aggregates)	[19,41,44,81,88]
Size Exclusion Chromatography (SEC)	Physical/Chemical—Columns packed with pore beads separate particles of various sizes and molecular weights	High yield Pure samples Maintains EVs intact	Potential contaminants (co-purifying protein aggregates) Samples can be diluted	[19,41,44,68,91]
Immunoaffinity Capture	Chemical—Uses antibodies to interact with specific membrane proteins	Quick High yield Pure samples	Expensive Lack of standardisation	[19,41,44,68,81,91]
Microfluidics	Physical/Chemical—Based on several principles including immunoaffinity, size and density	High yield Very pure samples	Expensive	[19,44,91]

**Table 3:** Commonly used hydrogel-based systems for the delivery of EVs.

Materials Used	Type of Cells/EVs	Applications	References
Hyaluronic Acid	MSC secretome	Asherman’s syndrome (injured endometrium)	[156]
Alginate	PPR exosomes/AD-MSC exosomes	Skin regeneration	[157,158]
Chitosan-based hydrogel with silk fibroin	hUC-MSC treated with miR-675-exosomes/gingival MSC exosomes	Aging-induced vascular disfunction/skin wound healing	[159,160]
Methylcellulose-chitosan	Placental MSC exosomes	Wound healing	[161]
Chitosan/chitosan-hyaluronic acid composite hydrogels	miR-126-3p overexpressing MSC exosomes/MSC exosomes/BM-MSC exosomes	Wound healing Ischaemia Skin regeneration	[162,163,164,165]
Hydroxyapatite embedded hyaluronic acid	UC-MSC exosomes	Bone regeneration	[166]
Polypetide-based FHE hydrogel	AD-MSC exosomes	Wound healing	[167]
RGD peptide/peptide-modified adhesive hydrogel	MSC exosomes	AKI repair/SCI treatment	[168,169]
Polyethylene glycol (PEG) hydrogel	MSC EVs	Chronic liver regeneration	[170]
Crosslinked hyaluronic acid/PEG hydrogel	MSC EVs	Osteoarthritis	[171]

Abbreviations: PPR, platelet rich plasma; hUC-MSC, human UC-MSC; FHE, pluronic F127, oxidative hyaluronic acid, and Poly-ε-L-lysine composite; AKI, acute kidney injury.

**Table 5:** Microcarrier culture methods used to increase the yield or enhance the properties of EVs.

Cellular Origin	Fold Increase	Characteristic Alteration	Reference
MSC (BM-, AD- and hUC-)	5.7-fold increase	Increased purity	[202]
hUC-MSC	18.38-fold increase	Increased inhibition of silica-induced PF	[213]
hUC-MSC	20-fold increase	More potent siRNA transfer to neurons	[214]
BM-MSC	24-fold increase	Increased neurite length in TG neurons	[215]
hSF-MSC	1.6-fold increase	NA	[216]
SHED	NA	Induce apoptosis in dopaminergic neurons	[217]

Abbreviations: SHED, human exfoliated deciduous teeth.

## Data Availability

Data sharing not applicable.
